# Presentation and evaluation of an atypical, supraclavicular mass in a pediatric patient

**DOI:** 10.1093/jscr/rjac340

**Published:** 2022-09-30

**Authors:** Austin D Schafer, David Z Allen, Weston L Niermeyer, Charles A Elmaraghy, Miriam Conces

**Affiliations:** The Ohio State University College of Medicine, Columbus, OH, USA; The Ohio State University College of Medicine, Columbus, OH, USA; The Ohio State University College of Medicine, Columbus, OH, USA; The Ohio State University College of Medicine, Columbus, OH, USA; Department of Pediatric Otolaryngology – Head and Neck Surgery, Nationwide Children’s Hospital, Columbus, OH, USA; Department of Pathology and Laboratory Medicine, Nationwide Children’s Hospital, Columbus, OH, USA

## Abstract

Although the vast majority of pediatric neck masses are benign, pediatric malignancies commonly present in the supraclavicular region. We present the case of a 4-year-old male who presented with a mass in the trapezius muscle with accompanying lymphadenopathy. An extensive work-up was performed to exclude malignancy, and the patient was ultimately diagnosed with a benign monocytic mass, which surgically excised. He has been doing well since surgery with no evidence of recurrence. A review of the literature revealed this case to be the first of its kind to be reported.

## INTRODUCTION

Although 80–90% of pediatric neck masses are benign, they are common and can be an ominous presentation of malignancy [1, 2]. Of the malignant neck masses, lymphomas and rhabdomyosarcomas are the most common [1]. The supraclavicular region in particular is known as a common place for such malignancies to develop [3]. After a thorough history and physical exam, conservative imaging techniques such as ultrasound are utilized. If the history, physical and ultrasound are suggestive of a noninfectious process, then a core biopsy of the mass is performed. In addition, open excisional or incisional biopsies may be necessary. If histological evaluation of the biopsy is indicative of a neoplastic process, appropriate metastatic work-up and staging is then completed.

In this case, we present a 4-year-old male with supraclavicular fullness and an intramuscular, superficial neck mass that was treated with surgical excision. After an extensive pathologic review revealed infiltrating monocytes and histiocytes, the mass was deemed to be benign and the work-up was completed.

## CASE REPORT

**Figure 1:**
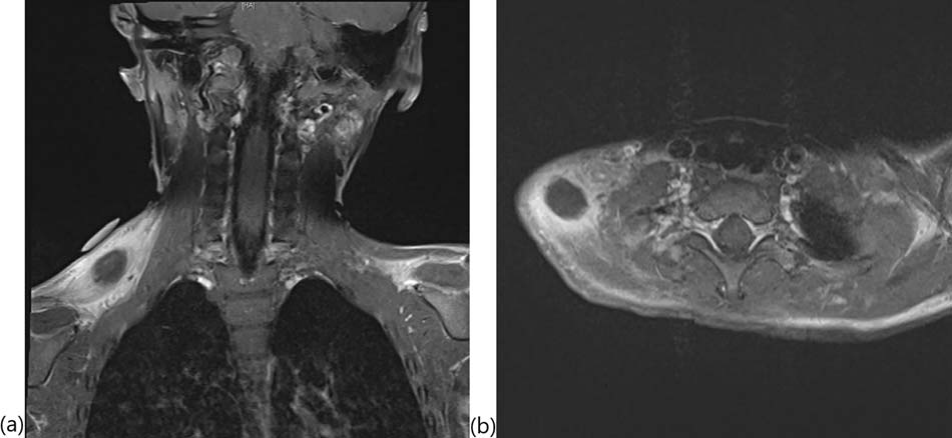
T1-weighted MRI with fat saturation showing the intramuscular mass in the (**a**) coronal and (**b**) axial planes.

A 4-year, 7-month-old male presented to our otolaryngology department with supraclavicular fullness and an enlarging, firm, right-sided 2 × 3-cm neck mass. At the initial visit, his vital signs were remarkable for an isolated fever of 101.6°F. A pertinent review of systems was negative per the patient’s parents. Possible environmental exposures included mice and bats as the patient lived on a farm. Past medical history was unremarkable. Physical examination demonstrated a mass located on the superior dorsal side of the right shoulder with a few mildly enlarged, mobile and cervical lymph nodes. An ultrasound of the mass was negative for abscess. The patient was started on ampicillin and sulbactam due to concern for infection; however, a pharyngeal swab and chest radiograph were negative. A magnetic resonance imaging (MRI) demonstrated the mass within the mid-belly of the right trapezius muscle and revealed significant inflammation and cellulitis of the subcutaneous tissue, as well as a small fluid collection in the supraclavicular space consistent with necrosis (Fig. 1). A computed tomography (CT)-guided core biopsy was then performed. Gram stain, cultures and polymerase chain reaction diagnostics were all negative. Extensive necrosis in the biopsy prevented adequate pathologic evaluation.

**Figure 2:**
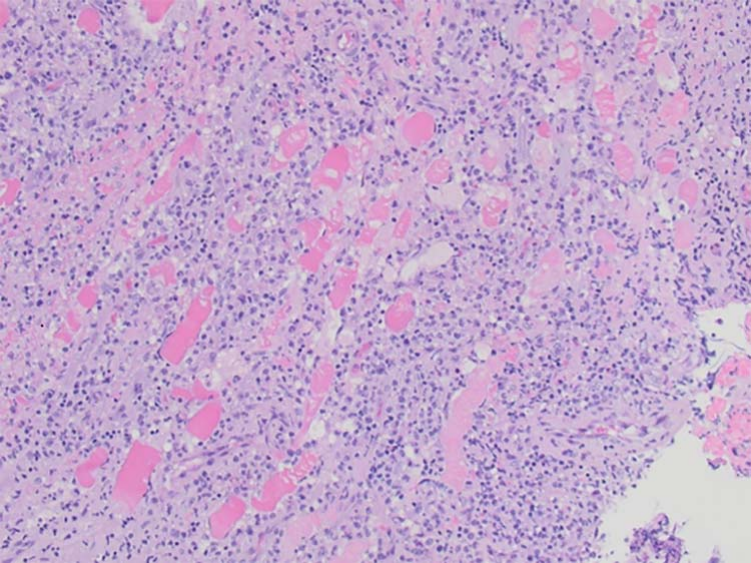
Skeletal muscle with a dense inflammatory infiltrate of predominantly histiocytes and monocytes (H&E, ×20 objective).

An excisional biopsy was performed 4 days after the initial biopsy, revealing skeletal muscle with a histiocytic/monocytic infiltrate with marked necrosis, and a lymph node demonstrating sinus histiocytosis (Fig. 2). Microscopic examination demonstrated a polymorphous inflammatory infiltrate within skeletal muscle and soft tissue. The infiltrate was predominantly composed of histiocytes and monocytes with scattered lymphocytes and neutrophils. Large regions of coagulative necrosis were present. A few large histiocytes showed atypical morphologic features such as vesicular chromatin and prominent nucleoli. However, upon immunohistochemical analysis, the cells did not express aberrant markers and showed a low proliferative index.

Anaplastic large cell lymphoma was deemed an unlikely as CD30/ALK cells were absent. In addition, despite the infiltration of monocytes, negative CD34, CD117 and low mitotic activity (low Ki-67 index) decreased suspicion for monocytic sarcoma. A CT scan was also performed for further evaluation, which demonstrated a potential paratracheal mediastinal lymph node with questionable clinical relevance. Flow cytometry of this lymph node was negative for myeloid neoplasm and lymphoma (Table 1). Further analysis of the pathologic slides by the National Institute of Health was consistent with the initial analysis. The Ohio State Wexner Medical Center performed cytogenetic analysis, which was within normal limits except for two non-clonal, abnormal cells with unknown significance.

**Table 1:** Flow cytometry performed on the involved right lymph node

**Cell type**	**%**
B cells (polyclonal)	47
T cells	44.4
NK cells	4.2
Myeloid cells	<1.5
CD30 cells	0

**Figure 3:**
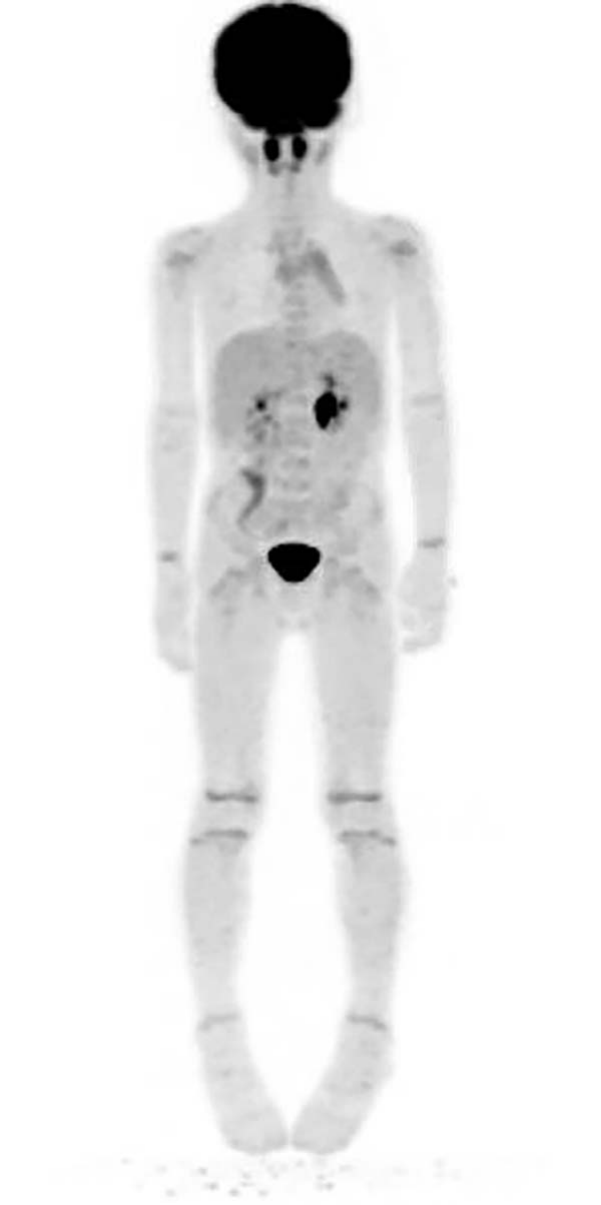
PET scan performed 4 months following surgical removal of the mass showing no abnormal uptake in the right neck/supraclavicular region.

Two weeks later, the patient reported no further symptoms at follow-up. However, due to the inconclusive pathology report, a bone marrow biopsy was scheduled to rule out a potential lymphoma. Twenty days following the clinic visit, the patient underwent another MRI as well as the bone marrow aspiration. This MRI demonstrated significant improvement in the size of the mass and no lymphadenopathy. The bone marrow biopsy demonstrated mild hypocellularity and nonmalignant characteristics (Table 2). Flow cytometry performed on this sample was negative for neoplasm (Table 3). A positron emission tomography (PET) scan completed 4 months from initial presentation demonstrated no increased uptake concerning for a neoplastic process (Fig. 3). The patient had no concerning symptoms and reported no further complaints on repeated follow-up.

**Table 2:** Relative cell percentages from bone marrow biopsy

**Cell type**	**%**
Blasts	0.2
Promyelocytes	0.8
Myelocytes	1.0
Metamyelocytes	0.4
Monocytes	0.8
Plasma cells	0.2

**Table 3:** Flow cytometry of the bone marrow aspirate

**Cell type**	**%**
Myeloblasts	0.5
Monocytes	3.2
Granulocytes	66.4
CD19+ cells	11.2
T cells	10.6 (CD4:CD8 1.2:1)
NK cells	1.5

## DISCUSSION

### Rationale for the diagnostic workup

Neck masses are common in pediatric otolaryngology and can be secondary to numerous etiologies. Although 80–90% of pediatric neck masses are benign, it is of the utmost importance to promptly rule out malignancy. Features indicative of a potential malignancy include firm and immobile lymph nodes with a supraclavicular location [2]. In this case, we describe a patient with a histiocytic/monocytic, infiltrating, supraclavicular mass within the trapezius muscle that, after an extensive work-up, was considered benign.

In general, posterior cervical space masses are typically lymphatic abnormalities consisting of cystic spaces and endothelial cells [3, 4]. In terms of malignant pathologies, cervical sporadic Burkitt lymphoma and cervical neuroblastoma are the two most common found in the perivertebral space [4]. Pilomatrixomas or cervical dermal sinuses are two benign lesions that could be found in this region [3].

The literature, however, is absent in regard to cases of benign, intramuscular masses presenting in children. This patient was extensively evaluated for a malignant process and neither excisional biopsy nor bone marrow biopsy led to a neoplastic etiology. There has been no recurrence since the excision. Although the etiology remains unclear, the patient’s environmental risk factors secondary to living on a farm make an insect, rodent or bat bite a potential source. In fact, the patient’s parents had specifically noted bats in the house previously. However, the patient did not present with any obvious skin lesions indicative of a bite, and the literature has not revealed any cases supporting this hypothesis.

## CONCLUSION

This is a case report of an initially aggressive supraclavicular neck mass in the trapezius muscle with monocytic/histiocytic pathology, which has not been previously described. This patient underwent extensive evaluation to rule out malignancy. Although the mass was ultimately deemed benign, we feel that this case demonstrates an example of a thorough work-up of an atypical pediatric neck mass.

## CONFLICT OF INTEREST STATEMENT

None declared.

## References

[1] Tracy TF Jr , Muratore CS. Management of common head and neck masses. Semin Pediatr Surg 2007;16:3–13. 17210478 10.1053/j.sempedsurg.2006.10.002

[2] Dickson PV , Davidoff AM. Malignant neoplasms of the head and neck. Semin Pediatr Surg 2006;15:92–8. 16616312 10.1053/j.sempedsurg.2006.02.006

[3] Jackson DL . Evaluation and management of pediatric neck masses: an otolaryngology perspective. Physician Assist Clin 2018;3:245–69. 32289087 10.1016/j.cpha.2017.12.003 PMC7140292

[4] Meuwly JY , Lepori D, Theumann N, Schnyder P, Etechami G, Hohlfeld J, et al. Multimodality imaging evaluation of the pediatric neck: techniques and spectrum of findings. Radiographics 2005;25:931–48. 16009816 10.1148/rg.254045142

